# Intragenic DNA methylation: implications of this epigenetic mechanism for cancer research

**DOI:** 10.1038/bjc.2011.550

**Published:** 2011-12-13

**Authors:** N Shenker, J M Flanagan

**Affiliations:** 1 Epigenetics Unit, Department of Surgery and Cancer, Hammersmith Hospital, Faculty of Medicine, Imperial College London, 4th Floor IRDB, Hammersmith Campus, Du Cane Road, London W12 0NN, UK

**Keywords:** epigenetics, DNA methylation, breast cancer, intragenic, gene-body

## Abstract

Epigenetics is the study of all mechanisms that regulate gene transcription and genome stability that are maintained throughout the cell division, but do not include the DNA sequence itself. The best-studied epigenetic mechanism to date is DNA methylation, where methyl groups are added to the cytosine base within cytosine–guanine dinucleotides (CpG sites). CpGs are frequently clustered in high density (CpG islands (CGIs)) at the promoter of over half of all genes. Current knowledge of transcriptional regulation by DNA methylation centres on its role at the promoter where unmethylated CGIs are present at most actively transcribed genes, whereas hypermethylation of the promoter results in gene repression. Over the last 5 years, research has gradually incorporated a broader understanding that methylation patterns across the gene (so-called intragenic or gene body methylation) may have a role in transcriptional regulation and efficiency. Numerous genome-wide DNA methylation profiling studies now support this notion, although whether DNA methylation patterns are a cause or consequence of other regulatory mechanisms is not yet clear. This review will examine the evidence for the function of intragenic methylation in gene transcription, and discuss the significance of this in carcinogenesis and for the future use of therapies targeted against DNA methylation.

One of the principal epigenetic mechanisms that governs the transcriptional regulation of genes is the methylation of CpG dinucleotides. DNA methylation at the promoter regions is known to be important in both development and human disease, including cancer. CpG sites tend to cluster in higher densities at promoter regions than throughout the rest of the genome, which has led to the definition of the term, CpG island (CGI; Bird, 1986). These promoter-associated CGIs tend to be unmethylated (Takai and Jones, 2002), whereas up to 80% of the total number of CpG sites in a genome are methylated. Furthermore, CpG sites are also found at significantly higher densities in gene-rich compared with gene-poor areas of the human chromosomes (Weber *et al*, 2005). DNA methylation at promoter CpG sites leads to the repression of gene expression by altering the conformation of DNA itself and local histone structures (Ng and Bird, 1999; Cedar and Bergman, 2009). Dogma states that this prevents the recruitment of the transcription complex scaffolding that can activate RNA polymerase II. However, this mechanism has only been thought to account for the transcriptional control in approximately half of all genes, and this figure may indeed be as low as a subset of 200 genes in any given cell type (Weber *et al*, 2005). It is possible that other unexplored functions of DNA methylation have additional roles in the transcriptional process of genes that do not contain a promoter-associated CGI.

In cancers, a substantial proportion of genes with promoter-associated CGIs become hypermethylated (Jones and Baylin, 2007). Promoter hypermethylation frequently silences tumour-suppressor genes in cancers. Indeed, profiling of specific sites or panels of sites that are aberrantly methylated within tumour cells are currently being investigated as biomarkers of early prediction and prognostication, where cancer-associated methylation can be detected in tumour biopsy samples, cell-free serum, urine and peritoneal fluid (Schulz and Goering, 2011). Furthermore, DNA methylation profiling can also be used to define novel tumour subgroups (Flanagan, 2011).

A further understanding of the epigenetic mechanisms that regulate gene transcription in pathological states such as cancer will be crucial in the development of novel and innovative therapies for these diseases. Already, promoter hypermethylation can be reversed by non-specific demethylating agents, such as decitabine and 5-azacytidine (5-aza), which have been approved in the therapy of several haematological malignancies (Fandy *et al*, 2007). Few clinical trials of demethylating agents in solid tumours have been performed. To date, the largest phase 1 study in solid tumours suggested that the doses required to produce the same effects in humans as in mice were tolerable (Appleton *et al*, 2007), but as yet solid tumour demethylating therapies have not been successful in humans. The first published phase 2a trial suggested that azacytidine could help to reverse the platinum-based drug resistance in the patients with ovarian cancer (Fu *et al*, 2011). Although the intention of demethylating agents is to reactivate promoters that are silenced by promoter hypermethylation, the entire genome could become demethylated, including intragenic sequences. Recent investigations have also suggested that 5-aza has broader effects than purely as a demethylating agent, including altering activating histone modifications to repressive marks, and expression changes independent of promoter methylation (Komashko *et al*, 2008). However, this study only examined promoter regions and further insights may be gained by investigating the intragenic effects of demethylating agents on both histone and DNA methylation. The temporal nature of these events also requires further investigation. Therefore, understanding the role of intragenic methylation (IGM) in transcription and in carcinogenesis is critical to understand the full consequences of chemically induced demethylation as a therapy.

## IGM in lower organisms

**Figure 1:**
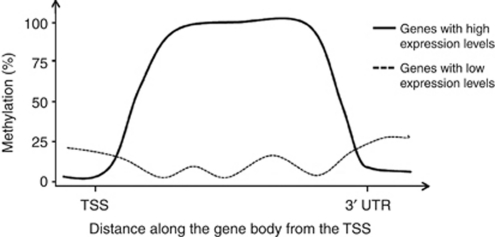
A schematic of the correlation determined by Zilberman *et al* for the methylation levels of differentially expressed genes. Abbreviations: TSS, transcription start site; 3′ UTR, three-prime untranslated region.

Clues regarding the role of IGM in a variety of organisms emerged over a decade ago (Yoder *et al*, 1997). The basic patterns of IGM are widely conserved throughout the plant and animal kingdoms (Feng *et al*, 2010; Zemach *et al*, 2010). In 2006, the first high-resolution genome-wide analysis of the methylation of CpG sites in *Arabidopsis thaliana* revealed that over one third of the genes had high levels of IGM, but only 5% of genes had high promoter region methylation (Zhang *et al*, 2006). A year later, Zilberman *et al* (2007) established a correlation between DNA methylation and the rate of gene transcription across the whole genome of *Arabidopsis*. Genes in the plant that were expressed at higher levels (in the top three deciles) tended to have higher levels of IGM than genes expressed at low levels, and all genes had low levels of methylation in the flanking regions (Figure 1). They hypothesised that the role of DNA methylation of the gene body was to suppress the unwanted expression of non-coding RNAs (ncRNAs) and the expression of transposons, which are epigenetically silenced in plants to reduce genomic instability. Frequently expressed genes would have higher levels of IGM in order to reduce the chance of aberrant ncRNAs being expressed or alternative intragenic promoters being active, in comparison with the genes that are expressed less frequently. The length of the gene was also important; shorter genes with higher levels of IGM levels are less likely to be expressed, whereas IGM appeared to have fewer transcriptional effects in longer genes. This may be principally because the effect of methylation on transcription is greatest at the extreme 5′ and 3′ ends of the gene.

## The role of IGM in humans

Several reports have now demonstrated a similar link in humans between the patterns of IGM and expression to those shown in lower organisms (Hellman and Chess, 2007; Ball *et al*, 2009; Rauch *et al*, 2009; Aran *et al*, 2011). In 2007, Hellman and Chess used an array approach to show the pattern of methylation across the X chromosome to investigate X-linked gene silencing. Contrary to their expectations, the active X allele was found to have higher levels of methylation than the silenced X allele, and 49% of the array probes were in intragenic coding regions of known genes. Furthermore, there was no bias towards methylation of repetitive elements in genes, which would have supported the hypothesis that the role of IGM is to suppress repetitive element transcription. Rauch *et al* (2009) used an enrichment technique for genome-wide methylated DNA to map methylation across the genome of human B cells. They confirmed that methylated intragenic regions correlated with higher levels of gene transcription, and IGM may be a mechanism that regulated the use of alternative promoters. This hypothesis gained supportive evidence from a study that examined DNA methylation in a specific genomic region in the human brain tissues, (Maunakea *et al*, 2010), discussed later in this review.

A further genome-wide study conducted in different human tissues showed a correlation between the rate of cell division between actively expressed and inactive genes and IGM (Aran *et al*, 2011), which mirrored the pattern previously found by Zilberman *et al* (2007). Furthermore, active and inactive genes in dormant tissue types, such as the brain and lung, were found to have unmethylated promoters and highly methylated intragenic regions, whereas methylation levels were reduced in inactive genes in rapidly dividing tissues such as the placenta and lymphocytes.

The precise definition of a promoter region is being redefined by new evidence that demonstrates the methylated state of the distal promoter region, and indeed extensions into the gene body, is relevant to the expression state of the gene. Appanah *et al* (2007) showed that the 3′ region of gene promoters had to be unmethylated to enable efficient transcriptional elongation. Recently, Hodges *et al* (2011) demonstrated hypomethylated regions (HMRs) that extended into the shore regions around promoters, as well as intragenic HMRs. Although the functions of these HMRs are unknown, the intragenic regions are associated with transcription factor binding sites, which suggest a biologically significant role.

The genomes of cancer cells also tend to show global hypomethylation alongside hypermethylation of the gene promoters. The latter has deleterious effects due to the silencing of tumour-suppressor genes and consequent acceleration in the oncogenic process. The causal mechanisms for genomic hypomethylation are still unknown, but a recent analysis suggests that it is unlikely to be due to a dysregulation in DNA repair mechanisms or members of the DNA methyltransferase (DNMT) enzyme family (Wild and Flanagan, 2010). The consequences of hypomethylation, whether intergenic or intragenic, are also unclear, but may act in a similar manner to that postulated in plant intragenic sequences, where demethylation leads to the reactivation of repressed repetitive elements, and potentially further genomic instability. Specific IGM sites have already been implicated in several human cancers. Salem *et al* studied a panel of cells from human bladder and colon cancers, and found a 550-bp-long HMR of the intragenic region of the *PAX6* gene, which mapped to exon 5. PAX6 is a highly conserved protein, which is important during embryogenesis and acts to stimulate uncontrolled cell growth. With the use of transfection studies, they showed that hypermethylation of exon 5 did not impede gene expression, but PAX6 protein levels fell when the promoter region was hypermethylated. In 2007, Smith *et al* (2007) identified aberrantly methylated areas within non-promoter CGI sites in the genes associated with colonic carcinoma, which appear to have a functional role by affecting mRNA transcription. These few studies have shown the value of investigating regions of DNA methylation in the areas outside of CGIs.

Data on the role and pattern of IGM in specific cancer types are currently limited. In breast cancer, which is the focus of our group's work, the distribution of hypomethylated CpG sites in primary tumours and cell lines has been shown to be clustered in gene-poor chromosomal regions, at the 5′ region of frequently expressed genes (including the promoter, first exon and first intron), and large intragenic regions of hypomethylation at chromosomal breakpoints, large genes and tissue-specific gene clusters (Shann *et al*, 2008). Further work that compares these data with normal tissue samples is required to determine the significance of this finding in cancer cells. We have previously shown significant variability in peripheral blood DNA methylation in the intragenic gene sequences in bilateral breast cancer cases compared with matched healthy control individuals using a tiled microarray (Flanagan *et al*, 2009). Most of the variability was found in intragenic repetitive elements, and one repetitive element in the *ATM* gene was associated with a three-fold increased risk of breast cancer in a larger case–control study. These data show potential biomarkers exist in the intragenic sequences of genes.

**Figure 2:**
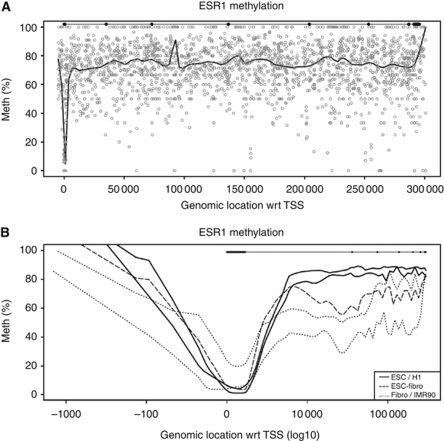
(**A**) Methylation levels of each CpG site (grey circles) along the full length of the *ESR1* gene from the transcription start site (indicated by ‘0’ on the *x* axis). The exons are shown along the uppermost horizontal line as full black circles. The black line indicates the smoothed average methylation levels across the gene. The data were obtained from the bisulphite sequencing study of peripheral mononuclear cells by Li *et al* (2010). (**B**) Genome-wide studies of human embryonic stem cells (ESCs, solid) and lung-derived fibroblasts (dotted) showed different patterns of IGM in the *ESR1* gene, and a broadly unmethylated promoter region. In ESCs that had differentiated into fibroblasts (dashed), the pattern of IGM bore a greater similarity to the adult differentiated pattern (IMR90 cell line, adult fibroblasts) than wild-type ESCs. The exons are shown along the uppermost horizontal line as full black circles, with *x* axis showing the log scale genomic location.

The first genome-wide sequencing study of a bisulphite-converted DNA sample from circulating white blood cells of a single individual was recently published (Li *et al*, 2010). These data, which is publically available, enable the genome-wide DNA methylation analysis of candidate genes of interest. Of particular interest in breast cancer is the *ESR1* gene, which encodes the oestrogen receptor alpha protein, which we have used to illustrate intragenic DNA methylation from whole-genome bisulphite sequencing data. The expression of ESR1 is of significant biological and prognostic importance in breast cancer, and largely drives the determination of the management plan. ESR1-positive tumours have broadly better outcomes, with slower-growing tumours on the whole, fewer metastases, and a greater range of hormonally targeted treatment options including tamoxifen and the aromatase inhibitors. Methylation data of the *ESR1* gene shows the typical pattern of methylation, with an unmethylated promoter and a highly methylated intragenic region (Figure 2A). Genome-wide bisulphite sequencing data have now been published for embryonic stem cells (H1 and hESCs), fibroblast cell lines (IMR90, fibroblasts) and ESCs induced to differentiate into fibroblasts (hESC-fibroblasts; GEO records: GSE19418 and GSE16256 (Figure 2B); http://www.ncbi.nlm.nih.gov/geo/). Information from further cell types will soon become available through projects such as the NIH Epigenomics Roadmap Project (http://www.roadmapepigenomics.org/). Further work is ongoing in our laboratory to discern the significance of the molecular mechanisms that underlie this pattern and its correlation with expression, and whether specific CpG sites across the *ESR1* gene may act as biomarkers. Although there are numerous ongoing studies for predictive and diagnostic biomarker CpG sites in the promoter region, the potential goldmine of information in intragenic regions has been relatively ignored to date.

## Postulated functions of intragenic DNA methylation: cause or consequence?

Given the conservation of methylation patterns across genes in both plants and animals (Suzuki and Bird, 2008; Feng *et al*, 2010), it is likely that gene-related DNA methylation is an ancient product of evolution that occurred before these kingdoms diverged, which is evidence of an important function. Early studies recognised the increased level of CpG in transposons, and postulated that the primary role of the methylation of repetitive elements was to suppress the expression of these parasitic insertions (Yoder *et al*, 1997). However, other mechanisms have now been proposed.

Intragenic CpG methylation may act to repress the initiation of transcription from alternative transcription start sites. Maunakea *et al* (2010) generated a CpG ‘map’, which included ∼88% of the genomic CpG sites, and then assessed the locations of methylated CpG sites in tissue from human brain tissue. CpG sites in intragenic, intergenic and promoter regions were found to overlap with sites of transcriptional initiation. The differential methylation levels of these three regions was postulated to correlate with the transcription of alternative isoforms in different tissue types, depending on the transcription start site that was unmethylated. However, the authors acknowledge that this genome-wide approach to the data analysis may prevent the recognition of smaller-scale regulatory mechanisms of DNA methylation. A similar mechanism that has been proposed relates to the modification of the transcriptional efficiency of RNA polymerase II and the transcriptional complex (Lorincz *et al*, 2004). This could be mediated by the modulation of the regional chromatin structure and may explain why there is a differential pattern of methylation in actively dividing tissues *vs* dormant tissues.

An alternative hypothesis is that methylation of the DNA along the sense strand could suppress the expression of antisense strand mRNA, either as full-length ncRNA transcripts or microRNAs, being initiated from a variety of intragenic sites. These antisense strands might interfere with the transcription of sense RNA due to their complementarity (Tufarelli *et al*, 2003), whereby higher levels of antisense copies lead to binding between the sense and antisense strands, reducing the amount of free sense mRNA and consequently reducing the protein translation, in a similar manner to microRNA-mediated gene regulation.

Any hypothesis that accounts for the effects of DNA methylation must take into account the effect of nucleosomes, the constituents of chromatin that package DNA. Nucleosomes consist of an octomer of four histone types (H2A, H2B, H3 and H4), which is wrapped around by a 147-bp DNA. Histone variants have variable affinities for different DNA sequence motifs, including methylated DNA (Segal and Widom, 2009). Histone modifications act locally to either repress or enable transcription. Such modifications include the methylation, acetylation and sumoylation of amino-acid residues on histone tails that extend out from the protein core. CpG density has been found to correlate with specific histone marks, which may have importance in the efficiency of the transcription complex across the gene (Lorincz *et al*, 2004; Lieb and Clarke, 2005).

The interaction between histone modifications and the intragenic DNA methylation, whether cause or consequence, is still unclear and currently being investigated. For example, the H3K36me3 histone mark is enriched at the regions of high CpG density (Hahn *et al*, 2011). H3K36me3 has been associated with hypermethylated DNA in the intragenic regions of actively expressed genes, where this mark may act to recruit DNMTs and maintain the methylated state (Hawkins *et al*, 2010). Intragenic H3K36 methylation has been shown in yeast to inhibit gene transcription from alternative start sites (Carrozza *et al*, 2005). There is some evidence of specific types of histones at exonic regions compared with intronic (Lieb and Clarke, 2005; Huff *et al*, 2010), including the H3K36me3 mark at the exons of highly expressed genes (Choi, 2010). Interestingly, Hahn *et al* (2011) indicated that the changes in intragenic DNA methylation levels did not affect the H3K36 histone modification locally and that the alternative reduction of H3K36 methylation by siRNA knockdown of SETD2 did not alter the intragenic DNA methylation. These data support the hypotheses that DNA methylation either occurs as a consequence of the chromatin environment only in some genes, or that two redundant and independent systems of gene repression have evolved. However, these data do not yet provide an answer to the question of cause or consequence, particularly at an individual gene level.

Certain types of histone modifications are associated with aberrant patterns of DNA methylation in cancer. Tri-methylated H3K9 (H3K9me3) and H3K27me3 are repressive histone modifications. H3K9me3 at the promoter region is associated with gene repression in plants, fungi and mammals, although the most recent evidence suggests that it does not affect the efficiency of transcription (Blahnik *et al*, 2011). The polycomb-associated repressive mark, H3K27me3, is found at aberrantly hypermethylated promoters in both cancer cell lines and tumour samples, where it appears to be preceded by an aberrant DNA methylation signature in inflamed tissues (Hahn *et al*, 2008). It may be that a form of epigenetic switching occurs at promoters to repress the gene transcription permanently, as DNA methylation replaces the more easily modifiable histone marks (Gal-Yam *et al*, 2008), but their interactions with DNA methylation across the gene body remain largely unexplored. Indeed, many of the commonly studied histone modifications at promoter regions, such as the activating mark, H3K4me3 that prevents DNA methylation at the promoter, have not been studied in depth across the gene body in tumour cells, although numerous studies have shown their relationship with the gene body in normal fetal and adult cell types (Guenther *et al*, 2007). As yet we can only speculate on the nature of the full interactions between histone modifications and DNA methylation in neoplastic states.

Nucleosome composition at the 5′ region of the gene, within the intragenic region, is of great importance in determining whether the gene is transcribed (reviewed in Lieb and Clarke (2005)). Typical patterns of methylation exist, such as the one shown in Figure 2, which demonstrate a steep increase in methylation levels within the first 8 kb from the transcription start site in actively expressed genes (Zilberman *et al*, 2007; Aran *et al*, 2011). Such patterns suggest that the first few nucleosomes in genes may be highly important for transcriptional efficiency. How this is affected by gene length and transcriptional activity remains unclear, but the recent publication by Han *et al* (2011) describes an exciting new strategy for the investigation of the temporal sequence of nucleosome occupancy, termed Nucleosome Occupancy Methylome Sequencing (NOME-Seq; Komashko and Farnham, 2010; Nanty *et al*, 2011). DNA methylation at the loci studied with NOME-Seq appears to occur after nucleosome positioning, and may be influenced by transcription factors, polycomb proteins and other chromatin remodelling factors. Choi *et al* (2009) also illustrated different levels of nucleosome occupancy and CpG density at the start and end of each exon in the majority of protein-coding genes, which may regulate the pausing of RNA polymerase II at these sites as it traverses the gene.

A recent study demonstrated a link between replication timing and IGM levels, where early replicating genes tended to be active and had higher levels of IGM (Aran *et al*, 2011). This study also showed a correlation between the rate of cell division between actively expressed and inactive genes, and IGM, which mirrored that pattern previously found by Zilberman *et al* (2007). Furthermore, active and inactive genes in dormant tissue types, such as the brain and lung, were found to have unmethylated promoters and highly methylated intragenic regions, whereas methylation levels were reduced in inactive genes in rapidly dividing tissues such as the placenta and lymphocytes. This preliminary data indicate a link between levels of intragenic DNA methylation and cell proliferation, but does not directly relate to the causality of increased proliferation in cancer cells.

In summary, there are several alternative hypotheses for the association between intragenic DNA methylation and transcription, including modifying transcription efficiency, altering the local histone conformation and producing different levels of sense and antisense mRNA. There is currently more data to support the hypothesis that intragenic DNA methylation is a consequence of other mechanisms of transcriptional regulation, including histone modifications, nucleosome positioning and replication timing. Nevertheless, DNA methylation remains an important marker of intragenic transcriptional efficiency and is experimentally easier to study. Interestingly, recent evidence suggested that variable methylation levels of individual CpG sites affect the binding affinity of transcription factors to nearby binding sites (Flower *et al*, 2010; Rishi *et al*, 2010). This may offer a further mechanism by which specific intragenic CpG sites can affect transcription.

## Future perspectives

Most of the work has focussed on CGI-associated methylation, as it is relatively easy to target short regions of DNA for investigation, such as promoter regions. Furthermore, the complexity and the cost of genome-wide, or even chromosomal-wide, studies of epigenetic mechanisms have been prohibitive until recently. With improvements in next generation whole genome bisulphite sequencing, including reduced costs and increased analysis speeds, new insights are being gained rapidly. As more cell types are bisulphite sequenced and published in freely available databases, and as more individuals are analysed, the challenges ahead will be in ensuring the depth of sequence reads and computational handling are optimised for the analysis of such high volumes of data. Only when these challenges are met will verifiable novel insights into the mechanisms of DNA methylation transcriptional control be possible. By studying CGI promoters exclusively, we have only scratched the surface of the epigenetic landscape of normal and cancer cells. Demethylating agents, including 5-azacytidine, are already being used in the management of haematological malignancies and myelodysplastic syndrome, and are in clinical trials for multiple solid tumours. The ability to target specifically aberrant regions of methylation in cancer cells holds promise for truly personalised therapies. However, a thorough understanding of the basic mechanisms of DNA methylation in transcriptional control, including the role of IGM, is critical to fully understand the consequences and side effects of such treatments.
